# Presence of Intestinal Parasites in Patients with Chronic Non-Communicable Diseases in Masaya (Nicaragua)

**DOI:** 10.3390/tropicalmed9080171

**Published:** 2024-07-30

**Authors:** Carla Muñoz-Antoli, Aleyda Pavón, Jacklyn Comas, Rafael Toledo, José Guillermo Esteban

**Affiliations:** 1 Área Parasitología, Departamento Farmacia y Tecnología Farmacéutica y Parasitología, Facultad Farmacia y Ciencias de la Alimentación, Universidad Valencia, Burjassot, 46100 Valencia, Spain; jacklyn.comas@alumni.uv.es (J.C.); rafael.toledo@uv.es (R.T.); jguillermo.esteban@uv.es (J.G.E.); 2 Centro de Investigaciones y Estudios de la Salud, Universidad Nacional Autónoma de Nicaragua, Managua 14172, Nicaragua; apavon@gmail.com; 3 Health and Community Research Group, Tropical Infectious Diseases Line, Universidad Tecnológica del Chocó Diego Luis Córdoba, Quibdo 270001, Colombia

**Keywords:** non-communicable diseases, intestinal parasites, Nicaragua

## Abstract

Aims: A cross-sectional study was conducted in Masaya (Nicaragua) to estimate the prevalence of intestinal parasite (IP) infections in patients with non-communicable diseases (NCDs) and to determine the associations between the types of NCDs and patients’ epidemiological characteristics of infection. Methods: A total of 157 preserved faecal samples were examined (direct wet mount, formalin/ethyl acetate concentration and modified Ziehl–Neelsen technique). Microscopically positive faecal sample identification was completed by conducting a molecular study. Results: The total prevalence of IP was 52% in NCD patients. Diabetic patients presented an IP prevalence of 42%. *Blastocystis* presented the highest prevalence (42%). A molecular analysis of *Giardia intestinalis* (prevalence of 1.3%) revealed 100% of sub-assemblage BIII and the *Entamoeba* complex (5%) was identified as *E. dispar*. *Blastocystis* ST1 appeared in 44% of those suffering from diabetes and ST3 in 66% of those suffering from hypertension, while ST2 only appeared in those suffering with several NCDs simultaneously. In diabetic patients, the risk of infection is associated with having pets (*p* = 0.021) and land-floor houses. The risk of infection appears to be statistically related (*p* = 0.019) in those with several NCDs having received a previous helminthic deworming treatment. Conclusions: Coordinated public health activities for IP and NCD screening and diagnosis are crucial to their successful control programmes.

## 1. Introduction

Non-communicable diseases (NCDs) are those diseases that have a long duration (more than 6 months), show a slow progression and are not transmitted from person to person. These NCDs are commonly associated with older age groups, despite evidence showing that 17 million NCD deaths occur before the age of 70 years. From these early deaths, 86% are estimated to occur in low- and middle-income countries (LMICs) [1,2,3,4]. In these countries, there is some evidence of links between specific infectious pathogens and a subsequent development of NCDs [4,5,6,7]. In fact, the 2030 Agenda for Sustainable Development recognises NCDs as a major challenge for sustainable development [1].

According to this, several infections might influence the course of many NCDs or, in contrast, several NCDs can be aggravated by the concurrent presence in the same individual of one (or more) infections. Intestinal parasites (IPs) constitute a major medical and public health problem in LMICs [8], frequently causing gastrointestinal complications, malnutrition, growth retardation and host metabolism disorders and playing a potential role in chronic metabolic diseases such as diabetes and other NCDs [9].

Currently, there are no epidemiological data on the prevalence of IPs in adults in Nicaragua, though a number of studies on children in Nicaragua indicate the high prevalence of a range of IPs [10,11,12,13,14,15]. The most prevalent protists (parasitic/commensal protozoa) species reported are *Blastocystis* (ranging from 45% to 77%), *Giardia intestinalis* (ranging from 17% to 45%) and other non-pathogen commensals such as *Entamoeba coli* (ranging from 21% to 41%). The most prevalent helminth species are *Trichuris trichiura* (ranging from 43% to 72%) and *Ascaris lumbricoides* (ranging from 20% to 36%) [10,11,12,13,14]. Coccidian were also reported with a *Cryptosporidium* prevalence of 35% [15].

According to the WHO’s NCD progress monitor [16], 76% of deaths were due to NCDs. In 2021, the most frequent NCDs reported in Nicaragua were hypertension, with a death rate of 3.5%, and diabetes mellitus type 2, with a death rate of 5.9% [17].

Herein, we conducted a cross-sectional study within Masaya (Nicaragua) to estimate the prevalence of IP infections in NCD patients. The aim of the present study was to report the prevalence of IPs in adults with NCDs and to determine the potential associations between the types of NCDs and patients’ epidemiological characteristics in relation to their infection. Furthermore, it was considered appropriate to perform a molecular approach of the most common IP detected in the NCD patient population studied, while trying to identify the circulating species and genotypes.

## 2. Materials and Methods

### 2.1. Study Design and Population

A cross-sectional study was conducted by enrolling adults suffering from NCDs attending the Alejandro Dávila Bolaños hospital (Masaya, Nicaragua) (Figure S1). The study was performed from October 2022 to January 2023. Masaya is located 28 km south of Managua. It has an estimated population of 22,826 inhabitants suffering NCDs for the year 2022 [16]. Knowing the reduction in faecal–oral infection routes after the COVID-19 pandemic, because of the increase in hygienic-sanitary habits, we estimated an IP prevalence rate of around 10%. A sample size calculation with the single proportion formula (https://www.calculator.net/sample-size-calculator.html?type=1&cl=95&ci=5&pp=50&ps=250&x=Calculate, accessed on 26 September 2022) showed that a sample of 138 was sufficient to estimate the prevalence of IP infections with 95% confidence and 5% margin of error. The enrolment criteria were as follows: (1) signed informed consent; (2) age ≥ 18 years; (3) existing diagnosis of NCD. The exclusion criterion was not being an outpatient.

### 2.2. Sample and Data Collection

Patients were provided with a stool collection bottle. A single stool sample per patient was collected. The filled stool containers were collected over the next two days in refrigerated containers and transported to the Nicaraguan laboratory within 12 h of collection. A small portion of each fresh faecal sample was transferred into a 2 mL microcentrifuge tube, and kept at −20 °C until molecular study in Valencia (Spain), and the rest was fixed with 10% formalin. At the time of sample collection, demographic data (age, sex, type of NCD suffered) and a basic structured (yes/no) epidemiological questionnaire (previous deworming treatment, having pets, use of latrines, land-floor houses) was completed by each participant.

### 2.3. Microscopic Analysis

Firstly, preserved faecal samples were examined using the direct wet mount technique, and secondly, 2 g faecal material was processed using an in-house formalin/ethyl acetate concentration technique for the presence of intestinal protozoa and helminths. Two drops of the sediment were analysed on a glass slide, covered with a cover-slip and viewed (at 100× and 400× magnification) under light microscopy. Modified Ziehl–Neelsen staining was carried out for detection and confirmation of coccidians (at 1000× magnification).

### 2.4. Molecular Study

DNA was extracted from *Giardia intestinalis*, *Blastocystis* or *Entamoeba* complex microscopy-positive faecal samples. Aliquots of 200 mg of frozen faecal material were weighed into sterile microcentrifuge tubes placed on ice. Genomic DNA extraction was performed using QIAamp DNA Stool Mini Kit (QIAGEN, Hilden, Germany) according to the manufacturer’s instructions. DNA was eluted in 200 µL buffer AE, purified in molecular grade water (200 µL) and stored at −20 °C.

In the case of *G. intestinalis*, a multilocus sequence typing scheme based on the amplification of partial sequences of the glutamate dehydrogenase (*gdh*) and ß-giardin (*bg*) genes were used for genotyping purposes [18]. A semi-nested-PCR protocol targeting a ~432 bp fragment of *gdh* was applied. PCR reactions were performed in a final volume of 25 μL including 5 μL of genomic DNA and 0.5 μM of the primer pairs GDHeF/GDHiR in the primary reaction and GDHiF/GDHiR in the secondary reaction. Cycling conditions were 3 min at 95 °C (initial denaturation step) followed by 35 cycles of 95 °C for 30 s, 55 °C for 30 s and 72 °C for 1 min, with a final extension of 72 °C for 7 min. A nested-PCR protocol was used to amplify a ~511 bp fragment of the *bg* gene of *G. intestinalis*. PCR reactions were conducted in a final volume of 25 μL consisting of 3 μL of genomic DNA and 0.4 μM of the primers pairs G7_F/G759_R in the primary reaction and G99_F/G609_R in the secondary reaction. Cycling parameters for the primary PCR reaction were an initial step of 95 °C for 7 min, followed by 35 cycles of 95 °C for 30 s, 65 °C for 30 s and 72 °C for 1 min with a final extension of 72 °C for 7 min. The same conditions were used in the secondary PCR except that the annealing temperature was 55 °C [18]. Negative controls (grade water instead of DNA template) were introduced in all PCR reactions.

The Central Service for Experimental Research Support (SCSIE, UV) performed the sequencing by capillary electrophoresis with BigDye^®^ Terminator Chemistry Applied Biosystems (Foster City, CA, USA) in both forward and reverse directions using the primers described for the PCRs and an automated sequencer ABI PRISM 3130 (Applied biosystems, Foster City, CA, USA). Raw sequencing data were viewed using the Chromas Lite version 2.1 sequence analysis programme (http://www.technelysium.com.au/chromas.html accessed on 28 July 2024). Generated DNA consensus sequences were aligned to appropriate reference sequences using the MEGA version 6 software application to identify *Giardia* assemblages/sub-assemblages [19].

For *Blastocystis* subtype analysis, we used the primers and PCR protocols described by Scicluna et al. [20]. Direct PCR amplification of *Blastocystis* was performed, using the barcoding region primers (BhRDr/RD5), targeting a 600 bp fragment of the small rRNA subunit (*SSU* rRNA). The 25 µL reaction mixture included the following: 5 µL template DNA; 0.5 µM of each primer; 3.5 mM MgCl_2_; 200 µM dNTPs; 1 U Taq DNA polymerase (Thermo Scientific, Waaltham, MA, USA); 1× Taq reaction buffer. PCR conditions consisted of 35 cycles of initial denaturation at 95 °C for 2 min, followed by denaturation at 94 °C for 30 s, annealing at 60 °C for 30 s, extension at 72 °C for 30 s, and final extension at 72 °C for 1 min, carried out in a C1000 MJ mini-thermal cycler. Subtypes were determined using the sequence query facility in the *Blastocystis* SequenceTyping website, available at http://pubmlst.org/blastocystis/ (accessed on 28 July 2024) [21].

Differential identification of *Entamoeba* complex was performed using a simple PCR protocol, described by Hamzah et al. [22], that targets the small rRNA subunit. The combination of four oligonucleotides (ENTAF; EhR; EdR; EmR) specifically generated products easily distinguishable of 166 bp for *E. histolytica* DNA, 752 bp for *E. dispar* DNA and 580 bp for *E. moshkovskii* DNA [23].

### 2.5. Data Analysis

Statistical analyses were performed using the PAST version 4.05 (https://past.en.lo4d.com/download, accessed on 28 July 2024). A statistical comparison of categorical variables was carried out with the X^2^ test, 2 × 2 contingency tables and univariate analysis, OR (95%CI) and significance levels, of all different variables. Furthermore, a correspondence analysis was applied as a descriptive multivariate statistical technique that helps to measure similarities between NCDs and the strength of their relationships with different variables. All results were considered significant if the *p*-value was <0.05.

## 3. Results

### 3.1. Description of the Population Studied

A total of 157 adults affected by different NCDs were included in our study (Table 1). Their main NCDs were diabetes, hypertension and asthma. Furthermore, other patients presented a combination of several NCDs together.

### 3.2. Microscopy Data

The total prevalence of IP infections detected was 52% (81/157) (Table 1). No significant statistical differences between sex or by age group were detected. 

The prevalence of the different IPs observed is shown in Table 2, classified by protists (parasitic/commensal protozoa) at 51% (80/157) and helminths at 0.6% (1/157). *Blastocystis* showed the highest IP prevalence at 42% (66/157). The prevalence of *Giardia intestinalis* reached 1.3% (2/157) and 5% (8/157) for the *Entamoeba histolytica/dispar* complex. Only one case of helminthic infection with *A. lumbricoides* was recorded. No coccidia were observed.

The proportion of different IP species detected in each of the NCDs reported is presented in Table 3. *Blastocystis* presented the highest prevalence in all the different NCD patients. *Giardia intestinalis* was only found to be associated with diabetes and hypertension. Only one case of *A lumbricoides* was found in this study, and this patient had several NCDs. Among the different NCDs, the infection frequency reached a maximum of 69% (9/13) in those suffering from asthma, with statistically significant differences (*p* = 0.018). However, the 2 × 2 analysis did not demonstrate statistically significant differences between those who presented with asthma and those with several NCDs (*p* = 0.833).

Among those patients infected with IPs, mono-parasitism (44%) (36/81) appears to have no statistical differences compared with poly-parasitism (56%) (45/81). In those with poly-parasitism, the highest prevalence was reached with two IPs (64%) (29/45), followed by 3 IPs (29%) (13/45). Only 7% (3/45) reached poly-parasitism with four IPs. The different prevalences of mono- and poly-parasitism are shown in (Table S1).

### 3.3. Epidemiological Data

The relationship between the prevalence of IP infections and the different epidemiological characteristics in all the different NCDs reported is shown in Table 4. The risk of infection appears to be statistically related only in those with several NCDs having received a previous helminthic deworming treatment (*p* = 0.019).

All the species of protists appeared to be associated with anti-helminthic deworming treatment and land-floor houses. Moreover, the only patient with *A. lumbricoides* was not treated with anthelmintic, and this could only be related to the use of latrines. It is worthy to note that *G. intestinalis* infection was highly associated with coexistence with pets.

In those with diabetes, the infection appears mainly in those having pets, along with statistically significant differences (*p* = 0.021). In those with asthma, the infection appears mainly in those using latrines (*p* = 0.001). Moreover, a statistical correspondence analysis indicates that infection in patients suffering from diabetes is associated with having pets and land-floor houses, while in those with hypertension, it is associated with the use of latrines (Figure S2).

### 3.4. Molecular Results

A molecular analysis of two *G. intestinalis*-positive patients revealed that 100% of sub-assemblage B III was identical in both sequences. In the case of the eight patients positive with the *Entamoeba histolytica/dispar* complex, 100% were identified as *Entamoeba dispar*.

We were able to subtype only 21.2% (14/66) of the *Blastocystis* infection detected. Three different subtypes have been detected: ST1 (64.3%) (9/14), ST2 (14.3%) (2/14) and ST3 (21.4%) (3/14). *Blastocystis* ST1 is the most frequent subtype, with statistically significant differences (*p* = 0.009) among patients with NCDs.

*Blastocystis* ST1 appeared in 44% (4/9) of patients suffering from diabetes. *Blastocystis* ST2 only appeared in those with several simultaneous NCDs and *Blastocystis* ST3 in 66% (2/3) of those suffering from hypertension.

## 4. Discussion

IP infections are among the most important infectious diseases in low-income countries, causing relevant important mortality and morbidity, especially in patients with a compromised immune system and/or chronic diseases. In this sense, several chronic NCDs significantly increase the risk of opportunistic IP infections that can be severe by themselves in these type of patients, or even aggravate their condition due to chronic NCDs [24]. Herein, we analysed the prevalence of IP infections in a population of NCD outpatients of Masaya (Nicaragua). Our results showed that the IP prevalence (52%) is higher than that observed in previous reports in populations with similar health problems (4.16% to 46.8%) [24,25,26,27].

Strikingly, most of the IP infections observed herein were due to protists. Only in one individual was a helminth infection detected, specifically by *A. lumbricoides*, despite the fact that in other areas of Nicaragua, it is a frequent helminth [10,11]. The low prevalence of helminth infections may be indicative of the usefulness of albendazole as an anti-helminthic deworming treatment [28]. Nevertheless, we had found a positive association between the anthelmintic treatment and infections with protists. A possible hypothesis to explain this fact would be that the treatment can induce a certain relaxation in the prophylactic measures by patients who can also feel protected against protist infections by the pharmacological treatment. This fact highlights the need for better information strategies in these population groups regarding the coverage of the treatments received and the maintenance of prophylactic and self-care measures.

IP infections were more frequent in patients who suffered several NCDs simultaneously; however, it is impossible to know if the infection leads to the NCDs or if particularities of the NCDs lead to the infection. Efforts to achieve universal health care in LMICs must focus on the infectious risks leading to NCDs, especially in areas with high rates of these infectious conditions, to ensure equitable progress in reducing the burden of NCDs [8,29]. Both hypertension and diabetes have been commonly associated with a higher prevalence of parasite infections in different countries. For example, the highest prevalence of *Schistosoma mansoni* was found in Malawi patients with hypertension (85%), followed by those with diabetes mellitus (42%) [30]. Despite the authors failing to find an association between *S. mansoni* infection and NCD syndromes, previous studies demonstrated a close relationship between *S. mansoni* and diabetes mellitus in China [31]. In our study from Nicaragua, patients with diabetes mellitus or hypertension showed an IP prevalence of 42%. This is markedly higher than those reported previously, in a recent systematic review and meta-analysis, in which a prevalence of 26.5% for IP was found in diabetic patients [9]. However, in another survey in Brazil, the overall frequency of IPs in diabetic individuals reached 64% [32].

Despite asthma being relatively rare in the developing world [33,34], its potential association with parasite infections, especially migratory helminths, is of importance due to the potential respiratory consequences that involves this type of infection. In the present work, asthma was only referred in 8% of the patients. The low prevalence of intestinal helminths detected in our study prevents us from deeper considerations, though infections with *A. lumbricoides* have been associated with a higher risk of suffering from asthma, probably in relation to allergic-type reactions [35,36,37]. In fact, control strategies for *A. lumbricoides* infections have been shown to significantly improved the clinical impact of asthma [36]. We only detected a single patient infected with *A. lumbricoides* who did not suffer from asthma. Moreover, in Masaya asthmatic patients, most of the IP infections detected were only due to non-pathogenic species. It is likely that periodic anthelmintic treatment campaigns are also contributing to the low prevalence of asthma in this region.

*Blastocystis* is a frequent infection in patients suffering from NCDs, such as AIDS, cancer, organ transplantation or haemodialysis, compromising their immune system [24,38,39]. Among the thirty-five nations comprising the American continent, *Blastocystis*-positive samples have been reported in thirteen countries, but the *Blastocystis* subtypes are only known in ten different countries, but not in Nicaragua [40]. To our knowledge, this is the first study regarding a molecular approach to *Blastocystis* subtypes in Nicaragua in humans with chronic NCDs. We have detected a total of three *Blastocystis* subtypes in our study (ST1 (64.3%), ST2 (14.3%) and ST3 (21.4%)), with a marked predominance of ST1. Our results strongly support that ST1 is the most widely distributed *Blastocystis* subtype in *Blastocystis*-positive humans in the Americas [40].

The low diversity of *Blastocystis* subtypes (three subtypes) in humans found in our study differs with that observed in other South American countries such as Colombia (seven subtypes), Brazil (six subtypes) and Bolivia (five subtypes) [40,41,42]. Despite the information that *Blastocystis* subtypes in Latin America are subject to specific regional features, these data may serve to suggest an epidemiological pattern. ST1 and ST2 develop low host specificity, suggesting that their occurrence in humans is probably related to zoonotic infections. In fact, these subtypes have been commonly found both in humans and a wide range of animals, including monkeys, cattle, chickens, pigs, dogs and non-human primates [43,44]. In contrast, ST3 is considered to be more anthroponotic, and human-to-human transmission constitutes the main route [41,45,46]. This subtype has only rarely been isolated from primates, pigs, dogs, cattle and rodents [47]. Accordingly, although the occurrence and diversity of *Blastocystis* in animals has not yet been studied in Nicaragua, our results suggest that in diabetic and several NCD patients, the main source of infection could be the zoonotic *Blastocystis* ST1 and ST2, while in those patients with hypertension, the main source of infection could be the anthroponotic *Blastocystis* ST3.

The importance of the zoonotic transmission of the IP species is reinforced by the genotyping data of *G. intestinalis*. In our study, 100% of the *G. intestinalis*-positive samples belonged to zoonotic sub-assemblage B III. There is epidemiological and molecular evidence supporting the zoonotic route of transmission of the *G. intestinalis* genotype B among humans and dogs living in the same community [48]. In the area studied, pets inhabiting land-floor houses is a very common feature, which certainly favours the zoonotic transmission of parasites such as *Blastocystis* or *G. intestinalis* and may explain the high prevalence detected in NCD patients.

Among the non-pathogenic commensal-like organisms, *E. coli* and *E. nana* showed the highest prevalence. It should be considered that the studied population is especially vulnerable, and therefore, these people must be taken into account both for their interests in relation to possible indicators of failure in prophylactic measures, and for the potential medical impact of these organisms on people with weakened health. Both of these parasite species are able to chronically survive within the host and without apparent damage. However, the human intestinal ecosystem is a complex ambient environment where non-virulent parasites can yield an important effect on the normal microbiota composition [49,50] and have an impact on the immune status of the host [51,52,53], even in the absence of any signs of intestinal damage, thus being able to complicate the NCD symptomatology.

The greatest limitations of the study are as follows: (1) only one single stool sample was collected per outpatient, which might affect the microscopy sensitivity as shedding resistance forms is intermittent; (2) the small number of samples we were able to be subtype prevents us from determining the diversity and frequency of *Blastocystis* ST circulating in Nicaragua; (3) given the nature of the study, there was no non-NCD group, so comparisons were not addressed. These limitations should be considered for possible future studies with the aim of achieving a vision on global relationships between IPs and NCDs.

In summary, our study has shown a high prevalence of IPs in a group of vulnerable people, such as NCD patients in Masaya (Nicaragua). It is likely that in cases of complications or even a worsening of symptoms, the existence of these IPs should be considered in NCD patients. NCDs and IPs in LIMCs share common features, such as long-term care needs and overlapping high-risk populations, so fundamental data on IP and NCD comorbidity in LIMCs populations are needed. Coordinated public health activities for IP and NCD screening and diagnostics are crucial to their successful control programmes.

## Figures and Tables

**Table 1:** Description of the population studied and prevalence of IP infections in patients in Masaya (Nicaragua).

		Total Population N = 157		Infected N = 81		Infection 52%
		n	% (CI95%)	n	% (CI95%)	*p*-Value
Sex	Male	56	35.6 (28.5–43.4)	26	32.1 (22.6–42.8)	0.425
	Female	101	64.3 (56.6–71.5)	55	67.9 (57.2–77.4)	
Age group (years)	27–59	53	33.8 (26.7–41.4)	33	40.7 (30.5–51.7)	0.116
	60–74	93	59.2 (51.4–66.7)	44	54.3 (43.4–64.9)	
	75–90	10	6.3 (3.3–11.1)	3	3.7 (0.9–9.7)	
	>90	1	0.6 (0.03–3.1)	1	1.2 (0.06–5.9)	
NCD	Diabetes	53	33.8 (26.7–41.4)	22	27.2 (18.3–37.6)	0.018
	Hypertension	45	28.7 (22–36.1)	19	23.5 (15.2–33.6)	
	Asthma	13	8.3 (4.7–13.4)	9	11.1 (5.6–19.4)	
	Several	46	29.3 (22.6–36.8)	31	38.3 (28.2–49.2)	

**Table 2:** Prevalence of the different IPs observed in patients from Masaya (Nicaragua).

	Total Population N = 157	
	n	% (CI95%)
Protists	80	51 (43.2–58.7)
*Blastocystis*	66	42 (34.5–49.9)
*Giardia intestinalis*	2	1.2 (0.2–4.1)
*Entamoeba coli*	34	22 (15.7–28.6)
*Endolimax nana*	32	20 (14.6–27.2)
*Entamoeba histolytica/dispar*	8	5 (2.3–9.4)
Helminths	1	0.6 (0.03–3.1)
*Ascaris lumbricoides*	1	0.6 (0.03–3.1)
Total Infected	81	52 (43.8–59.3)
Negative	76	48 (40.6–56.2)

**Table 3:** Prevalence of different IP species detected in each of the NCDs reported by patients in Masaya (Nicaragua).

		Diabetes N = 53	Hypertension N = 45			Asthma N = 13		Several N = 46
	n	% (CI95%)	n	% (CI95%)	n	% (CI95%)	n	% (CI95%)
Protists	22	42 (28.8–55.1)	19	42 (28.5–56.9)	9	69 (41.3–89.4)	30	65 (50.7–77.9)
*Blastocystis*	18	34 (22.2–47.4)	15	33 (20.8–47.9)	7	54 (27.4–78.7)	26	57 (42.0–70.2)
*Giardia intestinalis*	1	2 (0.1–8.9)	1	2 (0.1–10.5)	0		0	
*Entamoeba coli*	10	19 (10–31.1)	5	11 (4.2–22.9)	6	46 (21.3–72.6)	13	28 (16.7–42.5)
*Endolimax nana*	5	9 (3.5–19.7)	8	18 (8.6–30.9)	6	46 (21.3–72.6)	13	28 (16.7–42.5)
*Entamoeba histolytica/dispar*	2	4 (0.6–11.9)	4	9 (2.9–20.1)	0		2	4 (0.7–13.6)
Helminths	0		0		0		1	2 (0.1–10.3)
*Ascaris lumbricoides*	0		0		0		1	2 (0.1–10.3)
Total Infected	22	42 (28.8–55.1)	19	42 (28.5–56.9)	9	69 (41.3–89.4)	31	67 (52.9–79.7)
Negative	31	58 (44.9–71.1)	26	57 (43.1–71.5)	4	31 (10.6–58.7)	15	33 (20.3–47.1)

**Table 4:** Prevalence of IP infections and the different epidemiological characteristics in all the different NCDs reported (n = total; Inf. = infected; OR = Odd ratios; 95%CI = 95% confidence interval; pv = *p*-value).

		Diabetes				Hypertension				Asthma				Several			
		n	Inf.	OR (95% CI)	pv	n	Inf.	OR (95%CI)	pv	n	Inf.	OR (95%CI)	pv	n	Inf.	OR (95%CI)	pv
		53	22			45	19			13	9			46	31		
Pets	Yes No	41 12	18 4	1.31 (0.5–3.1)	0.748	37 8	13 6	0.46 (0.2–0.8)	0.093	3 10	1 8	0.41 (0.1–2.1)	0.410	34 12	24 7	1.21 (0.7–2.0)	0.674
Deworming treatment	Yes No	12 41	8 14	1.95 (1.0–3.5)	0.093	20 25	10 9	1.38 (0.7–2.7)	0.521	7 6	4 5	0.68 (0.3–1.4)	0.676	28 18	23 8	1.84 (1.0–3.1)	0.019
Latrines	Yes No	25 28	12 10	1.34 (0.7–2.5)	0.530	20 25	5 14	0.44 (0.1–1.0)	0.073	11 2	8 1	1.45 (0.3–6.2)	0.847	31 15	21 10	1.01 (0.6–1.5)	0.792
Land-floor houses	Yes No	27 26	11 11	0.96 (0.5–1.8)	0.870	20 25	7 12	0.72 (0.3–1.5)	0.566	1 12	1 8	1.50 (1.0–2.2)	0.664	32 14	22 9	1.06 (0.6–1.7)	0.964

## Data Availability

All research data supporting reported results can be provided by the corresponding author upon request.

## Supplementary Materials

The following supporting information can be downloaded at: https://www.mdpi.com/article/10.3390/tropicalmed9080171/s1, Figure S1: Geographic location where the samples were collected in Masaya (Nicaragua). Table S1: Mono- and poly-parasitism detected in patients with chronic non-communicable diseases in Masaya (Nicaragua). Absolute values of the main combinations between parasites detected (*Entamoeba* complex* = *Entamoeba histolytica/dispar*). Figure S2: Visual representation of correspondence analysis demonstrating the strength of the relationships between epidemiological characteristics and NCDs.
