# Supplementary material for: Presence of Intestinal Parasites in Patients with Chronic Non-Communicable Diseases in Masaya (Nicaragua)
Source: Trop Med Infect Dis. 2024 Jul 30;9(8):171. doi: 10.3390/tropicalmed9080171 (PMC11359410; doi:10.3390/tropicalmed9080171)
Supplement: Supplementary file 1 [file tropicalmed-09-00171-s001.zip › tropicalmed-3079461-supplementary.pdf]

Tropical Medicine and Infectious Disease

MDPI

Supplementary Materials

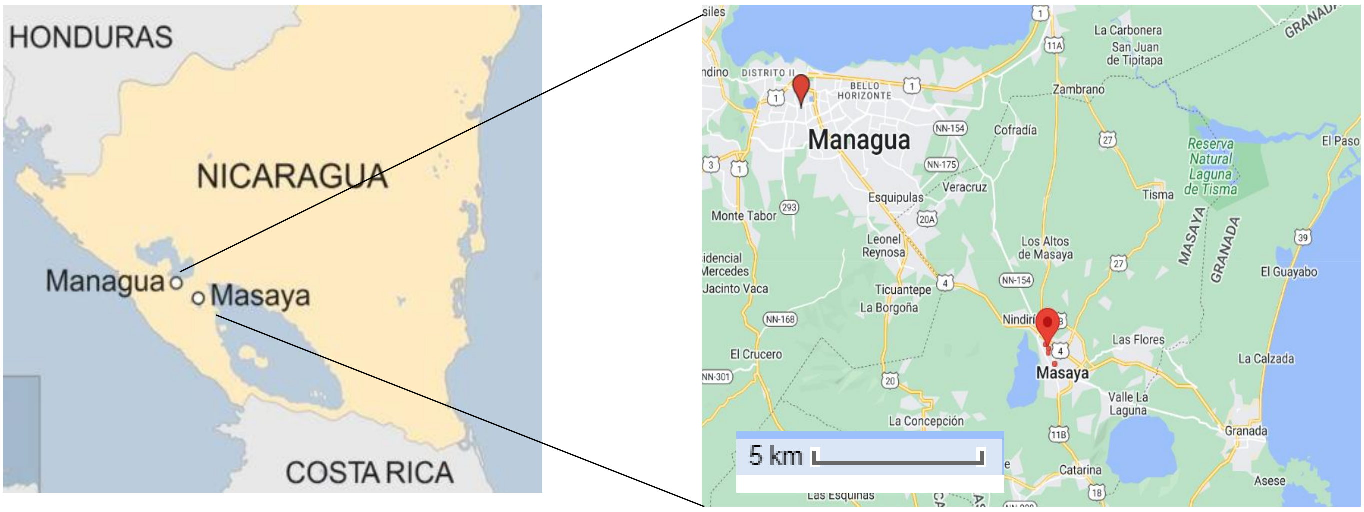

Figure S1. Geographic location where the samples were collected in Masaya (Nicaragua).

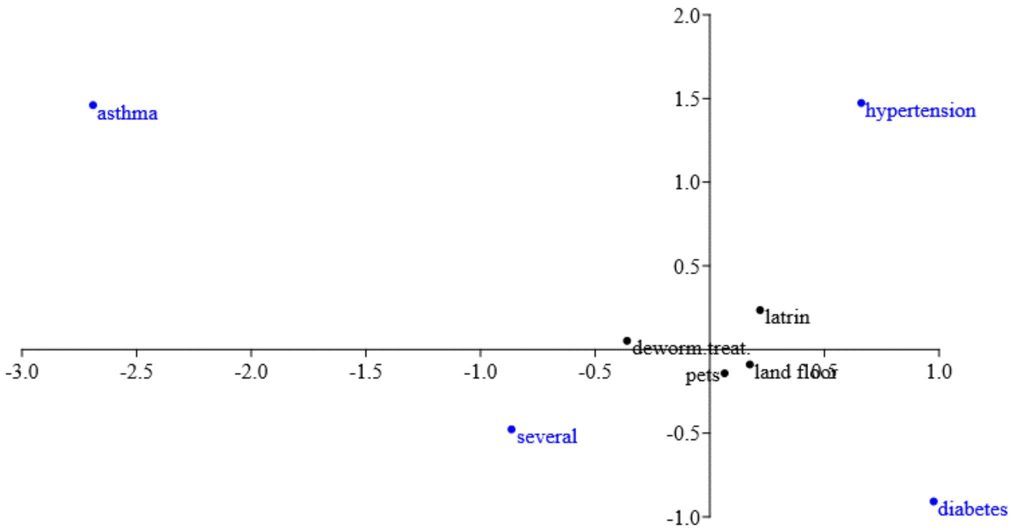

Figure S2. Visual representation of correspondence analysis demonstrating the strength of the relationships between epidemiological characteristics and NCDs.

Table S1. Mono- and poly-parasitism detected in patients with chronic non-communicable diseases in Masaya (Nicaragua). Absolute values of the main combinations between parasites detected (Entamoeba complex* = Entamoeba histolytica/dispar).

	Blastocystis	*G. intestinalis*	*E. coli*	*E. nana*	*Entamoeba* complex*	*A. lumbricoides*
Blastocystis	23	1	12	13	1	0
G. intestinalis	1	0	0	0	0	0
E. coli	12	0	8	1	1	0
E. nana	13	0	1	4	0	0
Entamoeba complex*	1	0	1	0	0	0
A.lumbricoides	0	0	0	0	0	1
Blastocystis + E. coli	-	0	-	10	1	0
Blastocystis + E. nana	-	0	0	-	2	0
Blastocystis + E. nana + Entamoeba complex*	1	1	2	-	-	0
